# Vitamin K supplementation does not prevent bone loss in ovariectomized Norway rats

**DOI:** 10.1186/1743-7075-9-12

**Published:** 2012-02-20

**Authors:** Xueyan Fu, Judith Moreines, Sarah L Booth

**Affiliations:** 1 Jean Mayer USDA Human Nutrition Research Center on Aging at Tufts University, 711 Washington Street, Boston, MA 02111, USA; 2 Pfizer Consumer Healthcare, 5 Giralda Farms, Madison, NJ 07940, USA

**Keywords:** Bone, Menaquinones, Phylloquinone, Vitamin K

## Abstract

**Background:**

Despite plausible biological mechanisms, the differential abilities of phylloquinone (PK) and menaquinones (MKn) to prevent bone loss remain controversial. The objective of the current study was to compare the effects of PK, menaquinone-4 (MK-4) and menaquinone-7 (MK-7) on the rate of bone loss in ovariectomized (OVX) Norway rats. A secondary aim was to compare the effects of vitamin K with those of bisphosphonates (BP) on bone loss.

**Methods:**

Rats (n = 96) were randomized to 6 dosing groups [n = 16/group; Sham; OVX; OVX + BP (100 μg/kg/100 μg/mL saline sc); OVX + PK; OVX + MK-4; and OVX + MK-7] for 6 wk. Equimolar daily doses of 107 mg PK/kg, 147 mg MK-4/kg, and 201 mg MK-7/kg diet were provided.

**Results:**

BP significantly increased bone strength and bone mineral density (BMD) *vs*. OVX (*P *< 0.05). However, PK, MK-4 or MK-7 did not change bone strength or BMD compared to the OVX group. Whereas supplementation of PK, MK-4 and MK-7 increased serum and tibia concentrations of each respective form, PK concentrations were consistently higher despite equimolar intakes.

**Conclusion:**

PK, MK-4, and MK-7 do not appear to prevent bone loss in OVX rats when administered concurrent with adequate intake of other nutrients.

## Background

There has been considerable recent interest in the role of vitamin K in bone metabolism [1-3]. Vitamin K is essential for the γ-carboxylation of specific glutamate residues in bone-specific proteins, including osteocalcin (OC). Without this modification, OC lacks the ability to bind to the mineral hydroxyapatite [4]. *In vitro *studies indicate that vitamin K can enhance bone mineralization and decrease bone resorption, either in its role as an enzyme cofactor or by mechanisms independent of γ-carboxylation [5,6]. For example, recent evidence suggests that vitamin K promotes the expression of osteoblastic markers through steroid and xenobiotic receptors (SXR)/pregnane X receptor (PXR)-modulated gene transcription [2,6].

Vitamin K exists in two primary forms in the diet: phylloquinone (PK) and menaquinones (MKn). PK, menaquinone-4 (MK-4) and menaquinone-7 (MK-7) have been collectively identified by various investigators as forms of vitamin K that have a potential role in preventing bone loss [7-10]. PK is the main dietary form of vitamin K, and is found in green leafy vegetables and vegetable oils [11]. MKn are primarily synthesized by bacteria in the gut, and differ from PK in the length of the side chain, which may contain 2-14 isoprenyl units at the 3-position of the naphthoquinone ring. MK-7 is present in traditional Japanese fermented foods (*natto*). MK-4 is unique in that it is converted from PK and menadione [12-15], although the exact mechanisms of conversion have yet to be elucidated.

Equivocal findings regarding efficacy of vitamin K on bone may be attributable to use of the different forms of vitamin K in both human and rodent studies. In Japan, MK-4 has been used in prevention and treatment of osteoporosis. Some studies have reported a positive effect of menaquinones on bone mineral content [16] and reduction in fracture risk [17]. However, most studies failed to show a consistent prevention effect of menaquinones in osteoporosis [18-20]. Although the individual effects of PK, MK-4 and MK-7 on bone metabolism has been reported, comparisons among all three forms have not.

The primary objective of this study was to compare the effects of three forms of vitamin K on prevention of bone loss in ovariectomized (OVX) female Norway rats. The three forms of vitamin K tested were PK, MK-4 and MK-7. Our secondary hypothesis focused on the comparative effects of different forms of vitamin K with those of bisphosphonates on bone loss.

## Materials and methods

### Chemicals

PK, MK-4 and MK-7 were provided by DSM, Eisai and J-Oil Mills Inc, respectively.

### Rats and diets

Rats were maintained and treated under protocols approved by the Institutional Animal Care and Use Committee (Bioanalytical Systems, Inc. Evansville). Ninety six unbred female rats (*Rattus norvegicus*, 20 wk) were obtained from Harlan (Indianapolis, IN), and acclimated at Bioanalytical Systems, Inc. (Evansville) one day post surgery prior to dosing. Animals were ranked by body weight, and maintained individually in stainless steel suspended wire cages to enable monitoring of food consumption and to minimize coprophagy. Animals were randomly assigned into 6 experimental groups (n = 16/group): Sham group (control animals who surgery to mimic an ovariectomy, but the ovaries are left intact); OVX control group; OVX+bisphosphonate (BP) group [100 μg/mL of saline (100 μg of Fosamax/kg body weight) daily by subcutaneous injection]; OVX+PK group (target: 0.3 mmol/kg diet; 135 mg/kg diet); OVX+MK-4 group (target: 0.3 mmol/kg diet; 133 mg/kg diet); and OVX+MK-7 group (target: 0.3 mmol/kg diet; 195 mg/kg diet). For 6 wk, all 6 groups were fed a low vitamin K diet (PK: 0.14 mg/kg diet, Harlan Teklad, TD.97053) [21] containing 1% calcium and 2.2 IU vitamin D/g, which were supplemented with one of the three different forms of vitamin K. Sham group rats were fed *ad libitum*. Amount of food offered for all OVX groups were adjusted weekly based on sham rats consumption. The food intake was monitored daily, and body weights were obtained weekly throughout the experiments.

Fasting blood was collected from all surviving animals at scheduled euthanasia by carbon dioxide inhalation, followed by exsanguination. Following processing, serum was stored at or below -70°C for subsequent analysis. Femurs, tibia, and lumbar spine (L1-5) were collected and stored at -20°C.

### Serum Ca and PO4 measurements

Ca and PO4 content of serum was determined by inductively coupled plasma optical emission spectrometry (ICP-OES, Optima 4300 DV, Perkin-Elmer), as described elsewhere [22].

### Bone strength

Right femurs and vertebrae were assessed biomechanically (breaking force and stiffness) and by measures of bone mineral density (bone calcium content). Bone strength was analyzed on the basis of a three-point bending test performed using a TA-ST2 Texture Analyzer (Texture Technologies Corp., Scarsdale, NY). To determine bone calcium content, femurs were dissolved overnight in 3 mL concentrated HNO_3 _and diluted to 25 mL using 1 mol/L HCl containing 0.5% La as LaCl_3_. From this, an appropriate dilution of each sample was prepared and the total calcium was assayed using atomic absorption spectrometry (AAnalyst 300 Perkin-Elmer, Inc., Norwalk, CT). Amount of calcium per gram of femur was calculated by dividing the amount of calcium in the femur by the weight of the femur.

### Vitamin K analysis

PK, MK-4 and MK-7 concentrations in the diet, serum and right tibia were determined by reversed-phase HPLC, as previous described [23]. The 2',3'-dihydrovitamin K was used as an internal standard for all analyses because of interfering peaks with K_(1,25) _usually used in assays.

### Statistical analysis

A natural log transformation was applied to serum and bone measures of PK, MK-4, and MK-7, to reduce skewness. The characteristics of the OVX and Sham groups were compared using an independent samples *t*-test. One-way ANOVA, followed by Tukey's Least Significant Difference for multiple comparisons, was used to compare the effect of BP with different forms of vitamin K on measures of bone strength and the serum and bone concentrations of vitamin K. All analyses were carried out using SPSS v. 14, and were considered statistically significant at *P *< 0.05.

## Results

### OVX compare to sham

Over the 6 wk study duration, average food intake was 15 g/d for all groups. As expected, all OVX groups had greater weight gain and lower bone strength compared to the Sham group (Table 1). OVX did not cause a significant alteration in serum or bone vitamin K concentrations as compared with that of Sham (data not shown). None of the rats had clinical signs of bleeding associated with vitamin K-deficiency.

**Table 1:** Body weight, bone biomarkers and characteristics of OVX control rats and their sham-operated controls fed low vitamin K diets for 6 wk^1,2^

	Sham	OVX
Body weight, g	273.6 ± 3.5	329.6 ± 3.6*

*Biochemical Measures*		

Serum Ca, mg/L	113.1 ± 1.1	114.3 ± 0.8

Serum PO4, mg/L	89.1 ± 2.5	90.9 ± 2.2

*Bone Strength*		

Ultimate Force, N	366.4 ± 24.2	231.8 ± 17.0*

Stiffness, Nmm	225.6 ± 20.4	163.9 ± 20.1*

Femur Ca, mg/g	122.2 ± 3.3	133.3 ± 1.8*

^1 ^Values are means ± SED, n = 16. ^2 ^Low vitamin K diet (Harlan Teklad, TD. 97053, PK: 0.14 mg/kg diet). * Different from Sham, *P *< 0.05

### Bone strength

As shown in Table 2, there was a significant increase in vertebral breaking force and stiffness in BP compared with OVX control. However, no effect of any of the vitamin K-supplemented groups on bone strength was observed. Bone mineral density (BMD), as assessed by average femoral calcium, was higher for the BP group compared with OVX. In contrast, BMD was not increased in any of the vitamin K-supplemented groups (Table 2).

**Table 2:** Bone characteristics of OVX rats fed with low or high vitamin K for 6 wk^1^

	Control^2^ PK:0.14 mg/kg diet	BP^2^ PK:0.14 mg/kg diet	PK 107 mg/kg diet	MK-4 147 mg/kg diet	MK-7 201 mg/kg diet
Body weight, g	329.6 ± 3.6	317.2 ± 4.2	331.9 ± 3.8	330.2 ± 3.9	338.3 ± 4.9

Ultimate Force, N	231.8 ± 17.0^a^	433.3 ± 21.0^c^	295.0 ± 15.0^ab^	245.8 ± 18.8^ab^	248.4 ± 16.2^ab^

Stiffness, Nmm	163.9 ± 20.1^a^	258.8 ± 30.2^b^	172.8 ± 17.1^a^	156.1 ± 19.4^a^	128.9 ± 10.9^a^

Femur Ca, mg/g	133.3 ± 1.8^a^	152.2 ± 2.2^c^	135.2 ± 3.1^a^	125.7 ± 2.4^b^	128.0 ± 1.4^ab^

Serum Ca, mg/L	114.3 ± 0.8^a^	108.1 ± 0.7^b^	112.3 ± 1.0^a^	113.8 ± 0.6^a^	113.0 ± 0.8^a^

Serum PO4, mg/L	90.9 ± 2.2^a^	75.8 ± 1.3^b^	87.2 ± 2.4^a^	86.9 ± 1.9^a^	88.2 ± 2.4^a^

^1 ^Values are means ± SED, n = 16. ^2 ^Low vitamin K diet (Harlan Teklad, TD. 97053). ^a,b,c ^When comparing measures across the five groups, means with different letter subscripts are statistically different, *P *< 0.05

### Vitamin K concentrations in serum and bone

Changes in serum and bone concentrations of different forms of vitamin K in response to supplementation are shown in Table 3. Based on direct HPLC analysis of the diets, the actual vitamin K contents of the diets were as follows: PK group (0.23 mmol/kg diet; 107 mg/kg diet); MK-4 group (0.33 mmol/kg diet; 147 mg/kg diet); and MK-7 group (0.31 mmol/kg diet; 201 mg/kg diet). At these doses of PK, MK-4 and MK-7, serum and bone PK concentrations were significantly greater compared to other forms of vitamin K. In MK-7 group, there was the unexpected finding of MK-7 epoxide in serum as determined by LC/mass spectrometry using an adaptation of the assay described elsewhere (data not shown) [24]. MK-7 epoxide was not detected in bone. In the absence of an internal standard for quantification, we can only acknowledge the presence of MK-7-epoxide in these samples.

**Table 3:** Vitamin K concentrations in serum and bone of OVX rats fed with low or high vitamin K for 6 wk^1^

	Control^2^ PK:0.14 mg/kg diet	BP^2^ PK:0.14 mg/kg diet	PK 107 mg/kg diet	MK-4 147 mg/kg diet	MK-7 201 mg/kg diet
**Serum**					

PK, nmol/L	0.2 ± 0.3^a^	0.1 ± 0.3^a^	238.0 ± 92.2^c^	0.0 ± 0.2^a^	1.9 ± 0.9^b^

MK-4, nmol/L	0.3 ± 0.5^a^	0.2 ± 0.5^a^	5.8 ± 2.5^b^	41.5 ± 17.7^d^	3.1 ± 1.0^c^

MK-7, nmol/L	ND^a^	ND^a^	ND^a^	ND^a^	31.4 ± 22.7^b^

**Tibia**					

PK, pmol/g	13.7 ± 12.5^a^	20.6 ± 14.0^ab^	1760.0 ± 581.0^c^	23.7 ± 21.1^ab^	13.4 ± 16.3^ab^

MK-4, pmol/g	ND^a^	ND^a^	ND^a^	62.4 ± 87.0^c^	1.0 ± 1.7^b^

MK-7, pmol/g	ND^a^	ND^a^	ND^a^	ND^a^	16.7 ± 14.0^b^

^1 ^Values are means ± SD, n = 16. ^2 ^Low vitamin K diet (Harlan Teklad, TD. 97053). ND: values were below the minimum detectable concentrations of 0.05 nmol/L or 0.05 pmol/g. ^a,b,c ^When comparing measures across the five groups, means with different letter subscripts are statistically different, *P *< 0.05

## Discussion

Vitamin K fed in one of three forms, PK, MK-4 and MK-7, for 6 weeks did not prevent bone loss in OVX Norway rats. These data lend support to the hypothesis that high doses of vitamin K do not have a preventive role in bone loss when administered concurrent to adequate amounts of other nutrients, including calcium and vitamin D.

Vitamin K has been studied in rodent models of bone loss, primarily in the form of MK-4 [9,25,26]. There is only one other report of a PK supplementation study in rats. In that study, PK also did not reduce bone loss following ovariectomy [27]. Although two studies reported no effect of MK-4 in the absence of vitamin D [28,29], the majority of studies have reported a positive effect of MK-4 on bone loss. Doses of MK-4 studied have ranged from 25 mg/kg diet to 480 mg/kg diet [28,29]. In this study, we used a dose of 147 mg/kg diet, which falls within this range of doses reporting positive effects. For comparison, the current recommended intake of any form of vitamin K in a rodent diet is 1 mg/kg diet [30]. That others have reported a positive effect of MK-4 on prevention of bone loss whereas there were no effects of MK-4 observed in the current study is an unexpected finding [25,27]. Increased concentrations of MK-4 in serum and the tibia provide confirmation that MK-4 was absorbed in this study, albeit with less efficiency than PK. Similarly, BP expectedly reduced bone loss, providing further evidence that the study design was suitable for measuring the effects of different compounds on prevention of bone loss following ovariectomy in these rats. Therefore we are currently unable to provide an explanation for our null findings. However the results of this rodent study are consistent with recent report of limited efficacy of MK-4 treatment on reduction of fraction risk in elderly men and women [20].

More recently, MK-7 has been reported to have greater efficacy in vitamin K functions compared to other forms. Of the two animal studies that have evaluated the role of MK-7 on prevention of bone loss following ovariectomy, both have reported beneficial effects [8,10]. The doses used in previous studies range from 94 μg/kg diet to 181 mg/kg diet, the latter being comparable to the 201 mg/kg MK-7 used in the current study. Moreover, Yamaguchi *et al. *reported that MK-7 had comparable beneficial effects on bone density when compared to equimolar doses of MK-4 [10]. In contrast, a recent clinical trial in children reported no effect of MK-7 supplementation on bone markers in children [31]. These conclusions are consistent with the findings of our rodent study.

An unexpected finding in the current study was the identification of MK-7 epoxide in the serum of the MK-7 animals. Vitamin K is recycled in the liver, with the epoxide form being an intermediate in the vitamin K cycle. The consistent appearance of the MK-7 epoxide in the serum suggests that the liver was unable to adequately metabolize the MK-7. The long-term implication of accumulation of MK-7-epoxide is not known. Unfortunately, we were unable to estimate the concentrations of MK-7 epoxide because we did not have a suitable internal standard. However this is an issue that merits further investigation prior to advocating high doses of MK-7 intake.

## Conclusions

In conclusion, supplementation of PK, MK-4 or MK-7 did not confer a beneficial effect on bone loss in ovariectomized Norway rats fed a diet that meets nutritional requirements for other nutrients, including calcium and vitamin D. This would suggest that equivocal findings in the literature regarding the effect of various forms of vitamin K on bone cannot be attributed to differences among the forms studied. These data are also consistent with a growing number of clinical studies that report no beneficial effect of vitamin K supplementation on bone loss in the elderly who are otherwise calcium and vitamin D-replete [1,18,19].

## Abbreviations

BMD: Bone mineral density; BP: Bisphosphonates; PK: Phylloquinone; MKn, Menaquinones; MK-4: Menaquinone-4; MK-7: Menaquinone-7; OC: Osteocalcin; OVX: Ovariectomized.

## Competing interests

X Fu and SL Booth declare that they have no competing interests. J Moreines is an employee of Pfizer Consumer Healthcare.

## Authors' contributions

All authors participated in the design of the research, analysis and interpretation of the data, and preparation of the manuscript. All authors read and approved the final manuscript.
